# Targeting JAK2/STAT3, NLRP3/Caspase-1, and PK2/PKR2 Pathways with Arbutin Ameliorates Lead Acetate-Induced Testicular Injury in Rats

**DOI:** 10.3390/ph17070909

**Published:** 2024-07-08

**Authors:** Hany H. Arab, Shuruq E. Alsufyani, Ahmed M. Ashour, Amany M. Gad, Alzahraa A. Elhemiely, Mohamed H. A. Gadelmawla, Marwa Ahmed Mahmoud, Ali Khames

**Affiliations:** 1 Department of Pharmacology and Toxicology, College of Pharmacy, Taif University, P.O. Box 11099, Taif 21944, Saudi Arabia; 2 Department of Pharmacology and Toxicology, College of Pharmacy, Umm Al Qura University, P.O. Box 13578, Makkah 21955, Saudi Arabia; 3 Department of Pharmacology and Toxicology, Faculty of Pharmacy, Sinai University, Kantara Branch, Ismailia 41636, Egypt; 4 Department of Pharmacology, Egyptian Drug Authority (EDA)—Formerly NODCAR, Giza 12654, Egypt; 5 Department of Histology, Faculty of Dentistry, Sinai University, Kantara Branch, Ismailia 41636, Egypt; 6 Department of Medical Physiology, Faculty of Medicine, Sohag University, Sohag 82511, Egypt; 7 Department of Pharmacology and Toxicology, Faculty of Pharmacy, Sohag University, Sohag 82511, Egypt

**Keywords:** lead acetate, arbutin, testicular toxicity, inflammation, oxidative stress

## Abstract

The reproductive system of males is adversely impacted by lead (Pb), a toxic heavy metal. The present study examined arbutin, a promising hydroquinone glycoside, for its potential ameliorative impact against Pb-induced testicular impairment in rats. The testicular injury was induced by the intraperitoneal administration of Pb acetate (20 mg/kg/day) for 10 consecutive days. Thirty-six rats were divided into six experimental groups (*n* = 6 per group): control, control treated with oral arbutin (250 mg/kg), control treated with intraperitoneal arbutin (75 mg/kg), untreated Pb, Pb treated with oral arbutin, and Pb treated with intraperitoneal arbutin. The treatments were administered daily for 10 days. Arbutin was administered by the oral and intraperitoneal routes to compare the efficacy of both routes in mitigating Pb acetate-induced testicular dysfunction. The current data revealed that both oral and intraperitoneal administration of arbutin significantly enhanced serum testosterone and sperm count/motility, indicating the amelioration of testicular dysfunction. In tandem, both routes lowered testicular histopathological aberrations and Johnsen’s damage scores. These favorable outcomes were driven by dampening testicular oxidative stress, evidenced by lowered lipid peroxidation and increased glutathione and catalase antioxidants. Moreover, arbutin lowered testicular p-JAK2 and p-STAT3 levels, confirming the inhibition of the JAK2/STAT3 pro-inflammatory pathway. In tandem, arbutin suppressed the testicular NLRP3/caspase-1/NF-B axis and augmented the cytoprotective PK2/PKR2 pathway. Notably, intraperitoneal arbutin at a lower dose prompted a more pronounced mitigation of Pb-induced testicular dysfunction compared to oral administration. In conclusion, arbutin ameliorates Pb-evoked testicular damage by stimulating testicular antioxidants and the PK2/PKR2 pathway and inhibiting the JAK2/STAT3 and NLRP3/caspase-1 pro-inflammatory pathways. Hence, arbutin may be used as an adjunct agent for mitigating Pb-induced testicular impairment.

## 1. Introduction

Human health is adversely affected by lead (Pb), a prevalent, nonbiodegradable pollutant [1]. The World Health Organization (WHO) remains concerned about exposure to Pb or its derivatives in polluted environments and occupational settings [2]. Among the sources of Pb pollution are pesticides, mining sites, industrial waste, vehicle emissions, and cosmetics [3,4,5]. In particular, occupational toxicity is regarded as a grim health concern linked to Pb toxicity, which poses a threat to human reproduction, especially in developing countries. Men’s infertility caused by exposure to environmental or occupational toxicants has received extensive attention in the past decade. Several industries present a risk of Pb exposure, including metal smelting, painting, glass-blowing, battery repair, and welding [1,4,6,7]. 

Pb is a highly pervasive xenobiotic metal pollutant that affects both humans and animals. It has a variety of toxicological effects on numerous organ systems, particularly the testis. Pb negatively impacts sperm viability and count and triggers alterations in sperm morphology, culminating in infertility [3,4,8]. There is still a great deal of uncertainty regarding the mechanisms underlying the reproductive toxicity of Pb. However, it is generally accepted that oxidative stress is one of the primary causes of Pb-induced testicular toxicity [3,7]. Moreover, Pb can inflict testicular damage by directly crossing the blood–testis barrier and injuring the testicular tissue or by disrupting the pituitary–testicular axis that regulates gonadal hormones. In the innate immune system, the pro-inflammatory inflammasomes have been implicated in the development and progression of testicular injuries. In this context, the nucleotide-binding domain, leucine-rich-containing family, pyrin domain-containing–3 (NLRP3) inflammasome is the most prevalent form of the inflammasome family [5]. There are three protein subunits that make up the NLRP3 inflammasome, including an effector protein called caspase 1, an adaptor protein called apoptosis-associated speck-like protein (ASC), and a sensor molecule called NLRP3 [5,7]. 

The Janus kinase/signal transducer and activator of transcription (JAK/STAT) is engaged in the majority of male reproductive processes, from the maturation of sperm to sexual development [9]. The formation and renewal of spermatogonial stem cells, which are necessary processes in male reproduction, as well as gametogenesis, have also been reported to be affected by JAK/STAT [9,10]. In the context of testicular pathologies, JAK2/STAT3 activation has been documented to trigger spermatogenic arrest and the diminished expression of the Sertoli cell junctions. Conversely, the inhibition of JAK2 has been reported to dampen oxidative stress and germ cell apoptosis, resulting in improved spermatogenesis integrity in experimental testicular pathology paradigms [9,11,12]. In the same regard, evidence reveals that the cytoprotective signal prokineticin 2 (PK2) is involved in several biological processes, including immune function, inflammation, angiogenesis, and nerve growth [13]. Moreover, the essential role of PK2 in gonadotropin-releasing hormones has been previously characterized. Mice with PK2 knockout suffer from deformed testes and impaired spermatogenesis [13,14]. Indeed, there are two functional complexes of prokineticin receptors (PKRs) in effector cells, namely, PKR1 and PKR2, which instigate PK2 biological effects. According to previous research, PKR2 plays a greater role in regulating testicular growth than PKR1 [13]. 

The rodent model of Pb-induced testicular injury has been characterized as a valuable tool for investigating agents with potential protective abilities on the male reproductive system [15,16]. In this regard, several phenolic compounds elicited favorable mitigation against Pb-induced testicular impairment and disrupted spermatogenesis. These compounds included epigallocatechin-3-gallate [3], luteolin [6], naringenin [4], hesperidin [16], and syringic acid [7]. Remarkably, this experimental model mimicked several pathological findings in humans, such as diminished sperm count and motility [4,6,16]. Moreover, the mechanisms that intercede testicular damage in rodents are similar to those impacting human subjects. These include an exaggerated pro-oxidant response, inflammation, and germ cell apoptosis [6,7,16]. 

**Figure 1:**
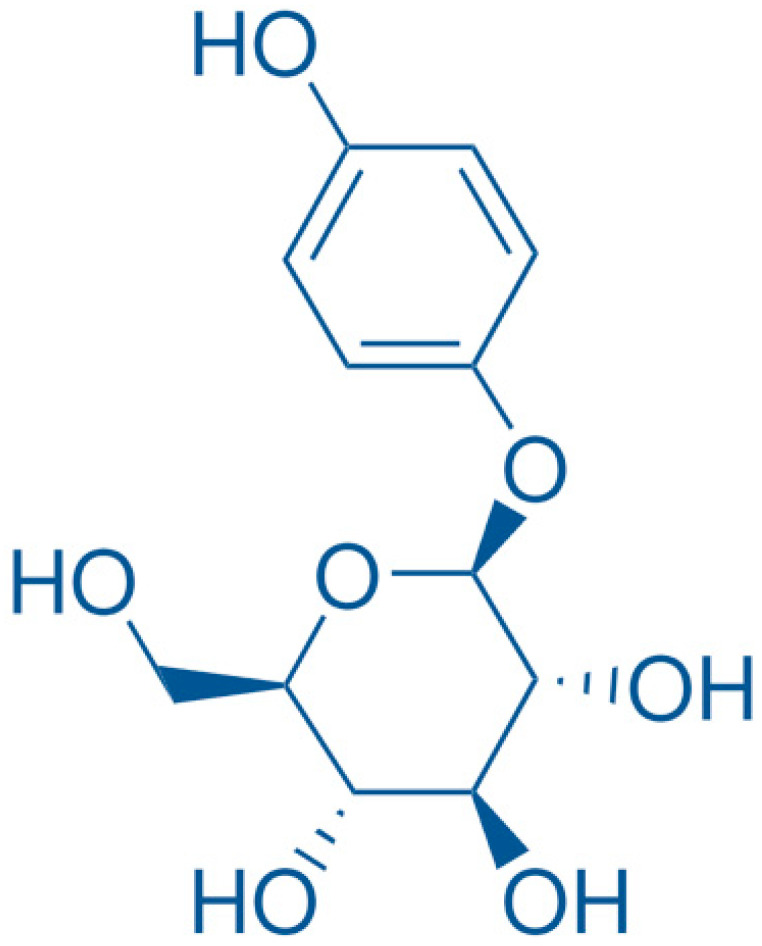
Arbutin’s chemical structure.

A significant increase in the use of natural products has taken place in recent years, and numerous lines of research have demonstrated the benefits of reactive oxygen species (ROS) scavengers and natural antioxidant polyphenols for dampening testicular injury. Arbutin (ARB; Figure 1), a bioactive polyphenol, is a glycosylated hydroquinone that naturally originates in various plant species such as the leaves of pear trees (*Rosaceae*), bearberry (*Ericaceae*), and *Bergenia crassifolia* [17]. Interestingly, ARB has also demonstrated marked anti-inflammatory [18,19] and antioxidant/radical-scavenging features [19,20,21]. Moreover, ARB has elicited an ameliorative impact against a rodent model of cyclophosphamide-triggered hepatotoxicity via combating oxidative stress, inflammation, and apoptosis [19]. In a mouse model of ulcerative colitis, ARB displayed marked competence in mitigating intestinal pathological findings through the suppression of myeloperoxidase and pro-inflammatory cytokines [18]. Curtailing inflammation and prooxidant events also mediate its favorable protection against cisplatin-evoked ovarian toxicity [21] and lipopolysaccharide-triggered acute kidney injury [22]. Yet, no previous report has examined the potential competence of ARB to dampen the toxic effects of Pb acetate on the testes in rats. The current study aimed to determine whether ARB can mitigate testicular dysfunction/impaired spermatogenesis. Particular focus was placed on antioxidant and anti-inflammatory mechanisms as potential targets underlying ARB’s protective effects against testicular impairment. In the present study design, ARB was administered by oral [23] and intraperitoneal [23,24,25] routes to compare the efficacy of both routes for mitigating Pb acetate-induced testicular dysfunction. In fact, the oral route has the advantage of being more convenient than intraperitoneal injection for animals [26]. Conversely, intraperitoneal administration exhibits higher bioavailability and minimal hepatic first-pass metabolism, curtailing adverse effects [23,27]. Evidence revealed that orally administered ARB is absorbed through the gastrointestinal tract, extensively metabolized in the human liver (but not in rodents), and excreted in the urine. According to human studies, ARB-rich diets have been found to increase plasma hydroquinone levels [26]. Conversely, experimental studies of rats have revealed that ARB excretion in urine remains unchanged after oral administration for 16 and 30 h [23]. 

In the current set of experiments, we employed a protective regimen for ARB administration, wherein ARB was co-administered with Pb. Considering the widespread presence of Pb in human industries such as metal smelting, painting, glass-blowing, battery repair, and welding [1,4,6,7], this protective approach was preferred to mimic human exposure conditions. Notably, this approach is in accordance with previous studies that investigated the efficacy of different agents in alleviating Pb-induced testicular dysfunction [6,24,25]. 

## 2. Results

### 2.1. Arbutin Improved Lead Acetate-Evoked Alterations in Semen Analyses

**Figure 2:**
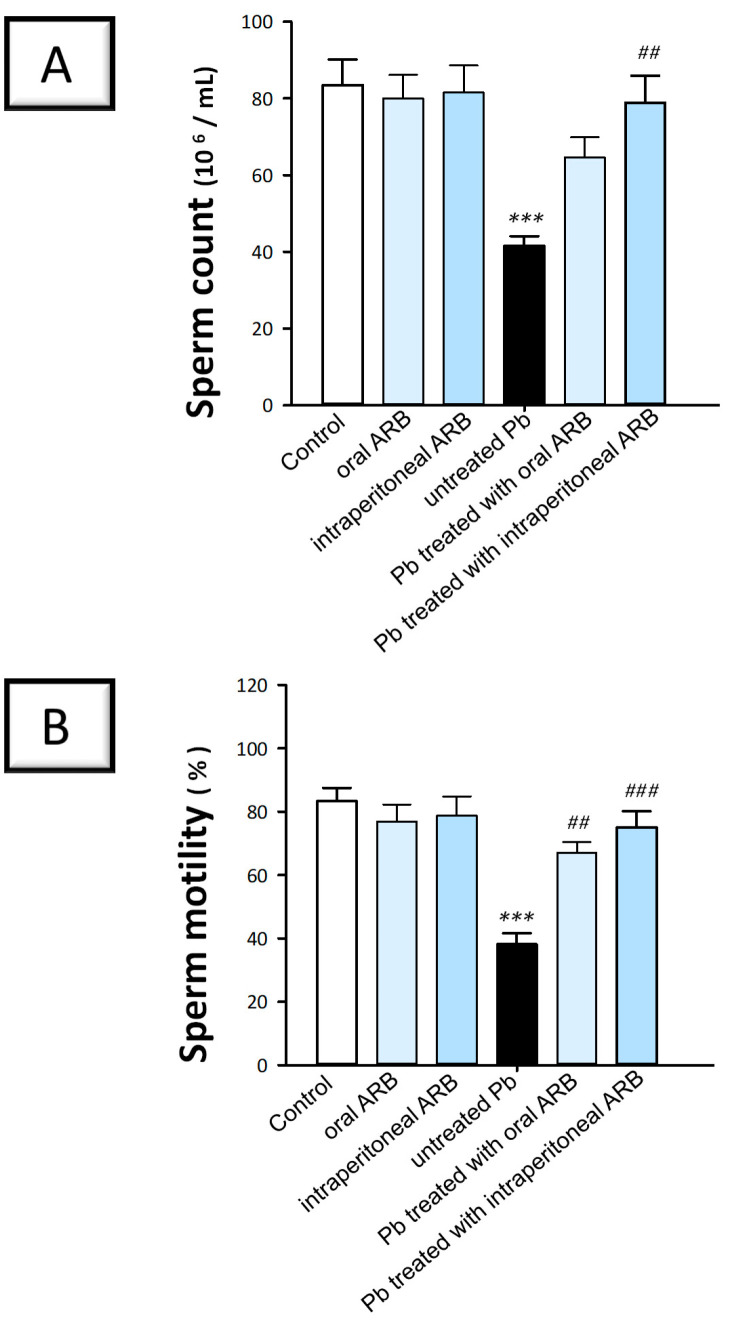

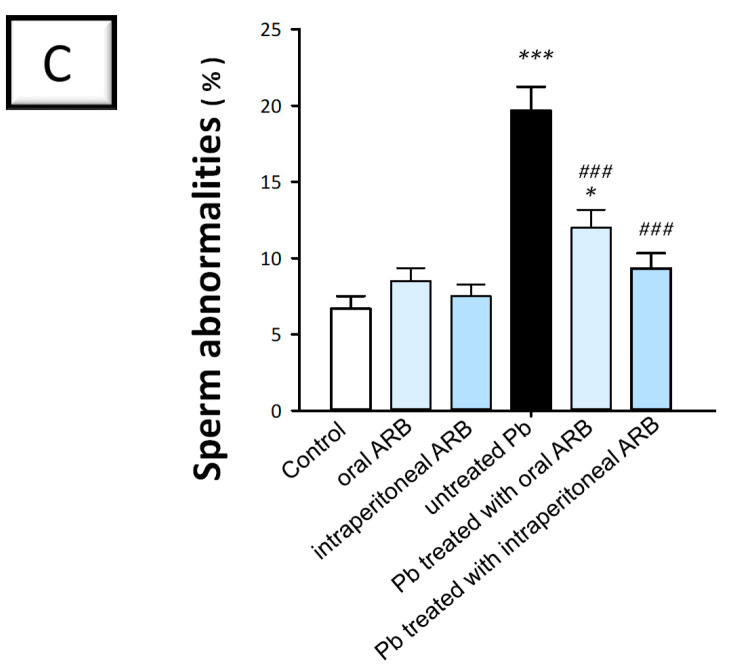
Ameliorative effect of arbutin (ARB), administered either orally or intraperitoneally, on sperm characteristics in lead (Pb)-evoked testicular damage in rats. ARB supplementation augmented sperm count (**A**) and sperm motility (**B**) while lowering sperm abnormalities (**C**) in the treated animals. Data represented as mean ± SEM (*n* = 6). Compared to the control group, significance was described by * *p* < 0.05 or *** *p* < 0.001. Compared to the untreated Pb group, significance was described by ^##^ *p* < 0.01 or ^###^ *p* < 0.001 (as determined by Bonferroni’s test and one-way ANOVA).

To investigate the potential protective effects of arbutin (ARB) using semen analyses of Pb acetate-intoxicated rats, we evaluated sperm count, sperm motility, and sperm abnormality. Sperm count has been widely recognized as an essential indicator of testicular function and the spermatogenesis process. Another indicator of testicular function is sperm motility, which reflects the sperm’s competence to navigate the female reproductive tract and fertilize an egg. Additionally, sperm abnormalities indicate morphological changes in sperm, denoting impaired spermatogenesis [28,29]. As shown in Figure 2A–C, Pb acetate intoxication significantly reduced epididymal spermatozoa count and motility by 50.3% (*p* < 0.001) and 54.2% (*p* < 0.001), respectively, and significantly elevated sperm cell abnormalities by 195% (*p* < 0.001) compared to the control group. On the other hand, the co-administration of ARB orally to Pb acetate-intoxicated animals reversed these effects. ARB significantly elevated sperm motility by 75.5% (*p* < 0.01) and significantly diminished sperm cell abnormalities by 39% (*p* < 0.001) compared to the Pb acetate group. Notably, the intraperitoneal injection of ARB to Pb acetate-intoxicated rats elicited a more pronounced mitigation of sperm characteristics than that of orally administered ARB. Specifically, intraperitoneal ARB boosted sperm count and motility by 89.9% (*p* < 0.01) and 96.9% (*p* < 0.001), respectively, while diminishing sperm cell abnormalities by 52.2% (*p* < 0.001) compared to the Pb acetate group. These findings reveal the ability of ARB to rescue spermatogenesis disruption triggered by Pb acetate in rats.

### 2.2. Arbutin Administration Rescued Serum Sex Hormones in Lead Acetate-Treated Rats

**Figure 3:**
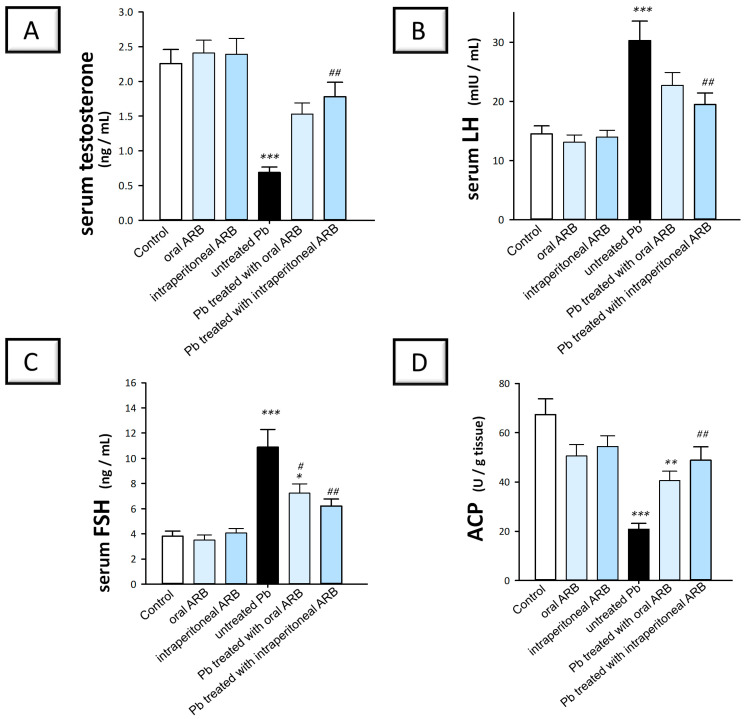
Ameliorative effect of arbutin (ARB) given orally or intraperitoneally on serum sex hormones in lead (Pb)-evoked testicular damage in rats. ARB boosted serum testosterone (**A**) and counteracted the increased serum LH (**B**) and FSH (**C**) levels. Moreover, ARB augmented testicular acid phosphatase activity in animals (**D**). Data represented as mean ± SEM (*n* = 6). Compared to the control group, significance was described by * *p* < 0.05, ** *p* < 0.01, or *** *p* < 0.001. Compared to the untreated Pb group, significance was described by ^#^ *p* < 0.05 or ^##^ *p* < 0.01 (as determined by Bonferroni’s test and one-way ANOVA).

To investigate the potential effects of ARB on testicular function and sex hormones in Pb acetate-intoxicated rats, we determined the serum levels of testosterone, LH, and FSH, together with the testicular ACP enzyme. Indeed, testosterone is the principal male sex hormone responsible for male reproductive organ growth, sexual function control, and spermatogenesis in the seminiferous tubules. Furthermore, LH, a gonadotropin released by the pituitary gland’s anterior lobe, is required for testosterone production via stimulating testicular Leydig cells [30,31]. Another gonadotropin released by the pituitary gland’s anterior lobe, FSH, works on Sertoli cells in the seminiferous tubules to promote spermatogenesis. Additionally, ACP, an enzyme found in testicular seminiferous epithelium, supports the sperm production process [30]. As illustrated in Figure 3A–D, Pb acetate-challenged rats showed a significant decrease in serum testosterone and testicular ACP activity by 69.6% (*p* < 0.001) and 69.2% (*p* < 0.001), respectively, along with a significant elevation in serum LH and FSH by 109%% (*p* < 0.001) and 185.5% (*p* < 0.001), respectively, compared to the control group. ARB administration by the oral route to Pb acetate-intoxicated animals counteracted these alterations, as seen by a significant elevation in testicular ACP activity by 95.6% (*p* < 0.01). This was accompanied by a significant decline in serum FSH by 33.5% (*p* < 0.05) compared to the Pb acetate-challenged group. Moreover, ARB administered by the intraperitoneal route afforded more pronounced changes in these parameters by significantly (*p* < 0.01) elevating serum testosterone and testicular ACP by 159.2% and 135.3%, respectively, as well as significantly (*p* < 0.01) lowering serum LH and FSH by 35.7% and 42.9%, respectively. These findings prove the capability of ARB to curtail Pb acetate-evoked testicular toxicity, likely by counteracting alterations in serum gonadal hormones and testicular ACP.

### 2.3. Arbutin Ameliorated Lead Acetate-Induced Histological Lesions in Testicular Tissue

**Figure 4:**
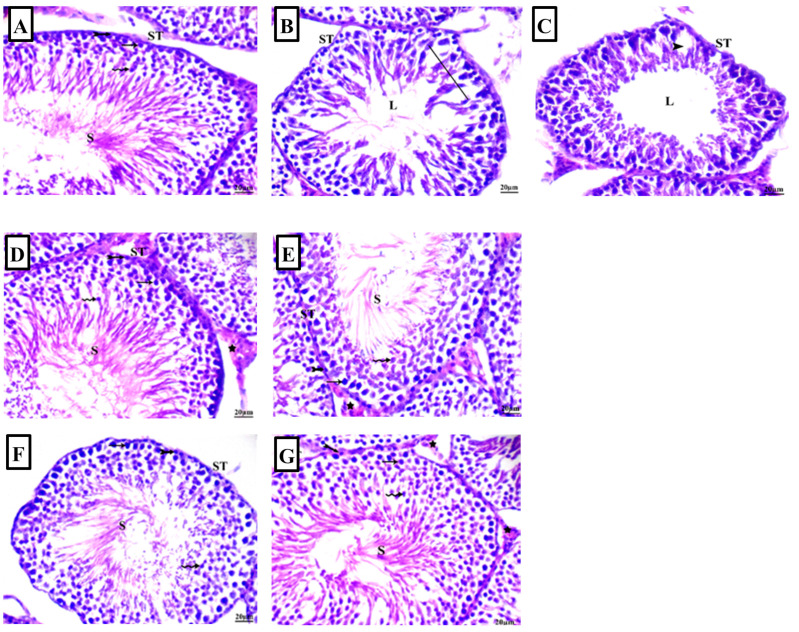

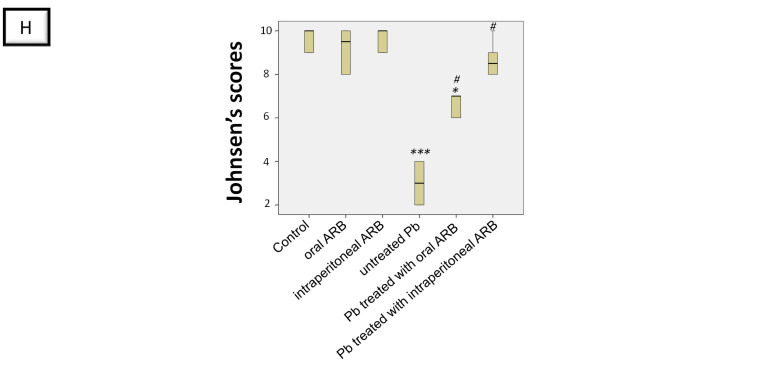
Photomicrographs of testicular tissue stained with hematoxylin and eosin (H-E) (scale bar: 20 µm). The photomicrographs are representative of n = 6 tissues. (**A**) In control animals, intact testicular architecture was observed with typical seminiferous tubules (STs) showing sperm (S), germinal cells (G), spermatocyte (thin arrow), spermatid (wavy arrow), spermatogonia (bifid arrow), and interstitial cells. Additionally, the interstitial space contained clusters of Leydig interstitial cells (**B**,**C**) Lead (Pb) acetate-challenged animals showed seminiferous tubule lumen lacking spermatozoa (L), a loss of germinal epithelium series (Line), and vacuolation (arrowhead). Intact testicular architecture with typical seminiferous tubules and intact interstitial cells (star) was shown in the control groups treated with oral (**D**) or intraperitoneal arbutin (ARB; (**E**)). In the groups co-treated with Pb and ARB via oral (**F**) or intraperitoneal injection (**G**), active seminiferous tubules and intact interstitial cells (star)were detected, implying attenuated testicular histopathological damage. (**H**) Spermatogenesis was scored according to Johnsen’s scoring scale. To present the scores, the medians and interquartile ranges are displayed (*n* = 6). The scores support the competence of oral and intraperitoneal administration of ARB to rescue the spermatogenesis process. Compared to the control group, significance was described by * *p* < 0.05 or *** *p* < 0.001. Compared to the untreated Pb group, significance was described by ^#^ *p* < 0.05.

To explore the potential effects of ARB on histopathological lesions triggered by Pb acetate, we used histopathology and quantified the extent of histopathological damage using Johnsen’s scoring scale for spermatogenesis. Johnsen’s scoring is a widely acknowledged quantitation tool for assessing the spermatogenesis process within the seminiferous tubules. The scoring scale ranges from 1 to 10, with higher scores reflecting normal spermatogenesis, while lower scores indicate impaired spermatogenesis [32]. An examination of H-E-stained testicular specimens revealed that control animals showed no histopathological alterations and had normal testicular architecture with typical seminiferous tubules. The seminiferous tubules showed a normal arrangement of spermatogenic cells, with spermatogonia located adjacent to the basement membrane and spermatocytes, spermatids, and spermatozoa progressing towards the lumen. The interstitial space contained clusters of Leydig interstitial cells (Figure 4A). Likewise, the administration of ARB to control animals by oral and intraperitoneal routes revealed intact histoarchitecture in the testicular tissues (Figure 4D,E, respectively). Conversely, the administration of Pb acetate showed disorganization of the seminiferous tubules, with disturbance in the spermatogenic cell sequence alongside a scarcity of sperm in most seminiferous tubules and the shedding of damaged spermatogenic cells inside the lumen of seminiferous tubules. Moreover, we observed diminished numbers of interstitial cells alongside the expansion/vacuolation of the interstitial spaces (Figure 4B,C). Interestingly, the co-administration of ARB by oral (Figure 4F) and intraperitoneal (Figure 4G) routes to Pb acetate-intoxicated animals improved the histological architecture in the testes of these animals. These improvements were further confirmed by Johnsen’s scoring scale for spermatogenesis within the seminiferous tubules (Figure 4H). In this regard, Pb acetate-challenged rats showed a significant reduction (*p* < 0.001) in spermatogenesis scores by 69% compared to the control animals. Interestingly, the oral and intraperitoneal administration of ARB to the Pb acetate-intoxicated animals instigated a significant elevation (*p* < 0.05) in Johnsen’s scores by 122.2% and 188.9%, respectively.

### 2.4. Arbutin Administration Mitigated Lead Acetate-Induced Oxidative Stress in the Testes of Rats

**Figure 5:**
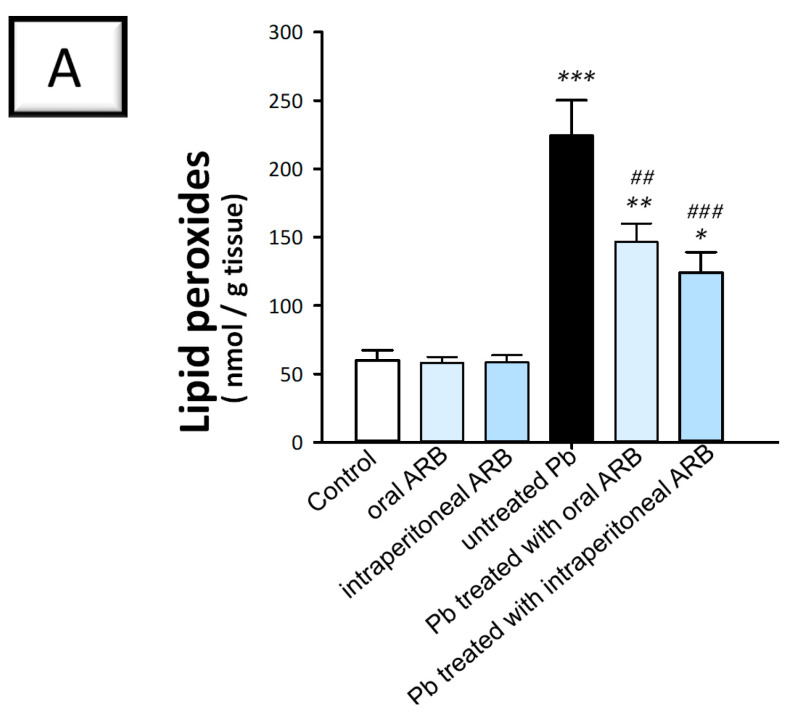

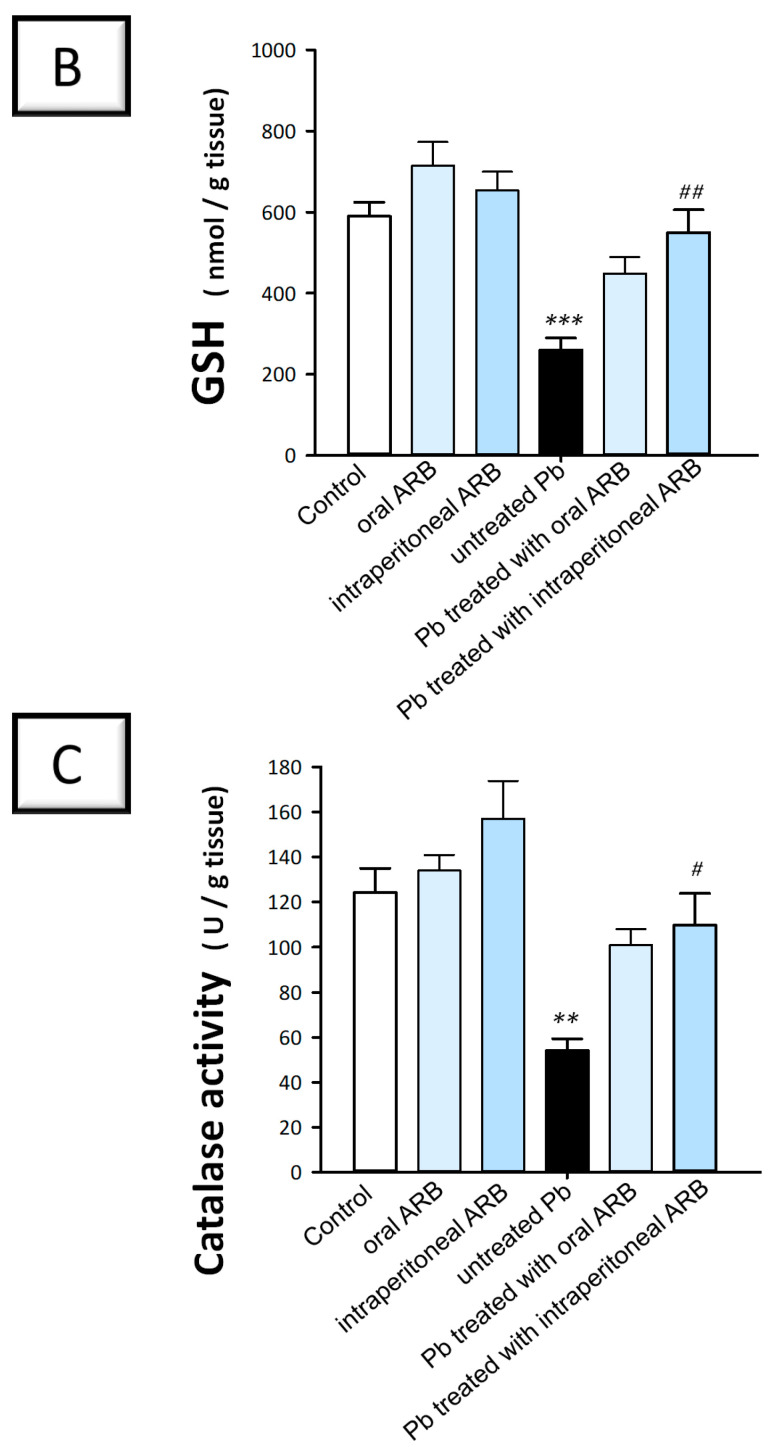
Mitigating effects of arbutin (ARB), administered either orally or via intraperitoneal injection, on testicular prooxidant/antioxidant events in lead (Pb)-evoked testicular damage in rats. ARB dampened the prooxidant lipid peroxides (**A**) and augmented reduced glutathione (GSH; (**B**)) and catalase activity (**C**) in the testicular tissues of animals. Data represented as mean ± SEM (n = 6). Compared to the control group, significance was described by * *p* < 0.05, ** *p* < 0.01, or *** *p* < 0.001. Compared to the untreated Pb group, significance was described by ^#^ *p* < 0.05, ^##^ *p* < 0.01, or ^###^ *p* < 0.001 (as determined by Bonferroni’s test and one-way ANOVA).

To examine the potential effects of ARB on Pb acetate-induced testicular oxidative stress, we measured the levels of lipid peroxides as well as the antioxidant signals of GSH and catalase. In the context of testicular oxidative stress markers, enhanced lipid peroxidation levels impair the histological structure and functional integrity of Leydig, Sertoli, and germ cells, culminating in diminished testosterone production and impaired spermatogenesis [4,6,33]. The depletion of testicular GSH may signal an impaired antioxidant defense system, denoting enhanced susceptibility to oxidative stress-induced damage and histopathological injury. In the same regard, diminished testicular catalase activity reflects an impaired competence to neutralize H_2_O_2_, resulting in an increased risk of testicular germ cell damage [34]. As shown in Figure 5A–C, the administration of Pb acetate significantly increased the levels of testicular lipid peroxides by 274.3% (*p* < 0.001) and significantly diminished testicular GSH contents and catalase activity by 55.9% (*p* < 0.001) and 56.5 % (*p* < 0.01), respectively, compared to the control group. The co-administration of ARB via the oral route to the Pb acetate group significantly decreased lipid peroxide content by 34.6% (*p* < 0.01) compared to the Pb acetate group. Moreover, ARB administered intraperitoneally demonstrated a more pronounced effect in mitigating oxidative stress by significantly increasing GSH (*p <* 0.01) and catalase (*p* < 0.05) by 111.3% and 103%, respectively, while significantly reducing lipid peroxide levels (*p* < 0.001) by 44.6% compared to the Pb acetate group. According to these findings, ARB’s inhibition of oxidative stress was, at least partially, responsible for the amelioration of Pb acetate-evoked testicular damage. 

### 2.5. Arbutin Suppressed the JAK2/STAT3 Pathway in the Testicular Tissue of Lead Acetate-Intoxicated Rats

**Figure 6:**
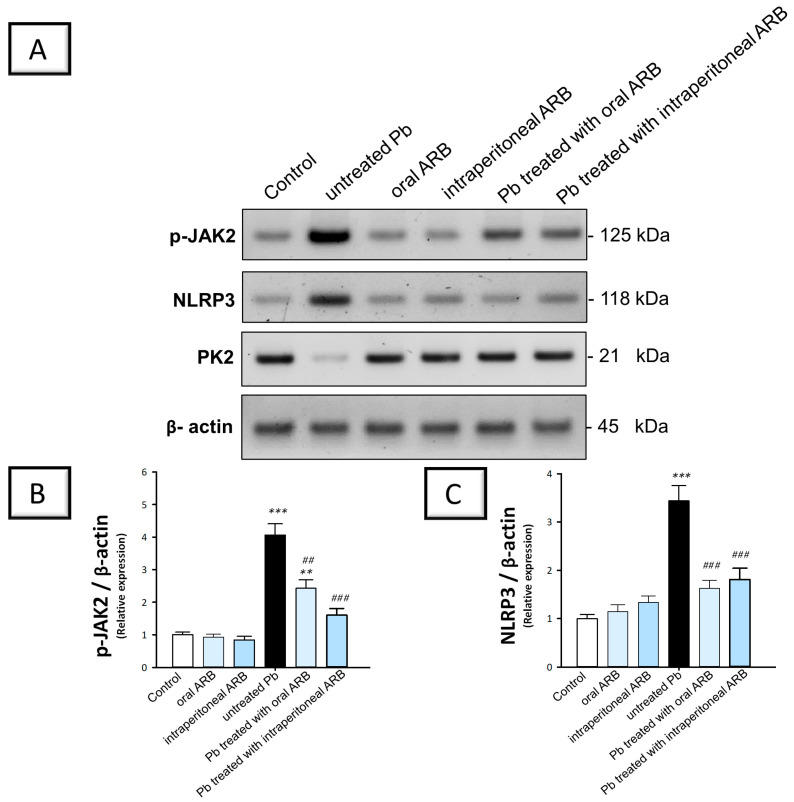

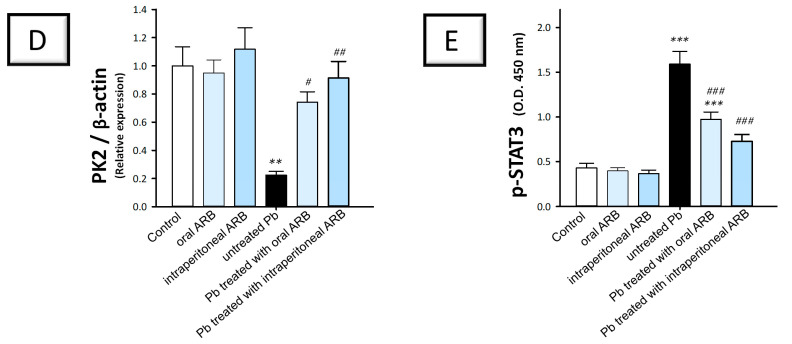
Modulatory effect of arbutin (ARB), administered either orally or via intraperitoneal injection, on testicular inflammatory signals in lead (Pb)-evoked testicular damage in rats. (**A**) Immunoblot analysis demonstrated the protein expression of p-JAK2, NLRP3, and PK2. To verify that total protein lysates were equally loaded, beta-actin was utilized as the loading control. (**B**) p-JAK2 quantification. (**C**) NLRP3 quantification. (**D**) PK2 quantification. In the Western blotting experiment, 1.0 was set as the control value. Data for the values in (**B**–**D**) are shown as mean ± SEM (from 3 independent experiments). (**E**) ARB lowers p-STAT3 in the testicular tissues of rats challenged with Pb. Data representation as mean ± SEM (n = 6). Compared to the control group, significance was described by ** *p* < 0.01, or *** *p* < 0.001. Compared to the untreated Pb group: significance was described by ^#^ *p* < 0.05, ^##^ *p* < 0.01, or ^###^ *p* < 0.001 (as determined by Bonferroni’s test and one-way ANOVA).

To explore the potential effects of ARB on the Pb acetate-induced activation of the pro-inflammatory JAK2/STAT3 pathway, we measured the levels of p-JAK2 and p-STAT3 in the testicular tissues. The JAK2/STAT3 pathway is essential for multiple testicular processes, including spermatogenesis and Sertoli cell function [9]. The stimulation of the JAK2/STAT3 pathway triggers an enhanced transcription of several genes involved in inflammation, oxidative stress, and apoptosis, resulting in detrimental effects on testicular function [9,12]. As illustrated in Figure 6B,E, rats challenged with Pb acetate showed a significant increase in testicular p-JAK2 and p-STAT3 pro-inflammatory signals by 305.4% (*p* < 0.001) and 271.6 % (*p* < 0.001), respectively, compared to control animals. The co-administration of ARB orally to Pb acetate-intoxicated animals triggered a significant decline in p-JAK2 and p-STAT3 protein expression by 40.3% (*p* < 0.01) and 38.8% (*p* < 0.001), respectively. ARB administered by the intraperitoneal route demonstrated a more pronounced suppression of p-JAK2 and p-STAT3, as seen by the significant reduction in p-JAK2 and p-STAT3 protein expression by 60.7% and 54.4%, respectively, compared to the Pb acetate group. According to these findings, ARB’s suppression of the JAK2/STAT3 pathway was, at least partially, responsible for the amelioration of Pb acetate-evoked testicular damage. 

### 2.6. Arbutin Curtailed the Testicular NLRP3/Caspase-1/NF-κB Pathway in Lead Acetate-Intoxicated Rats

**Figure 7:**
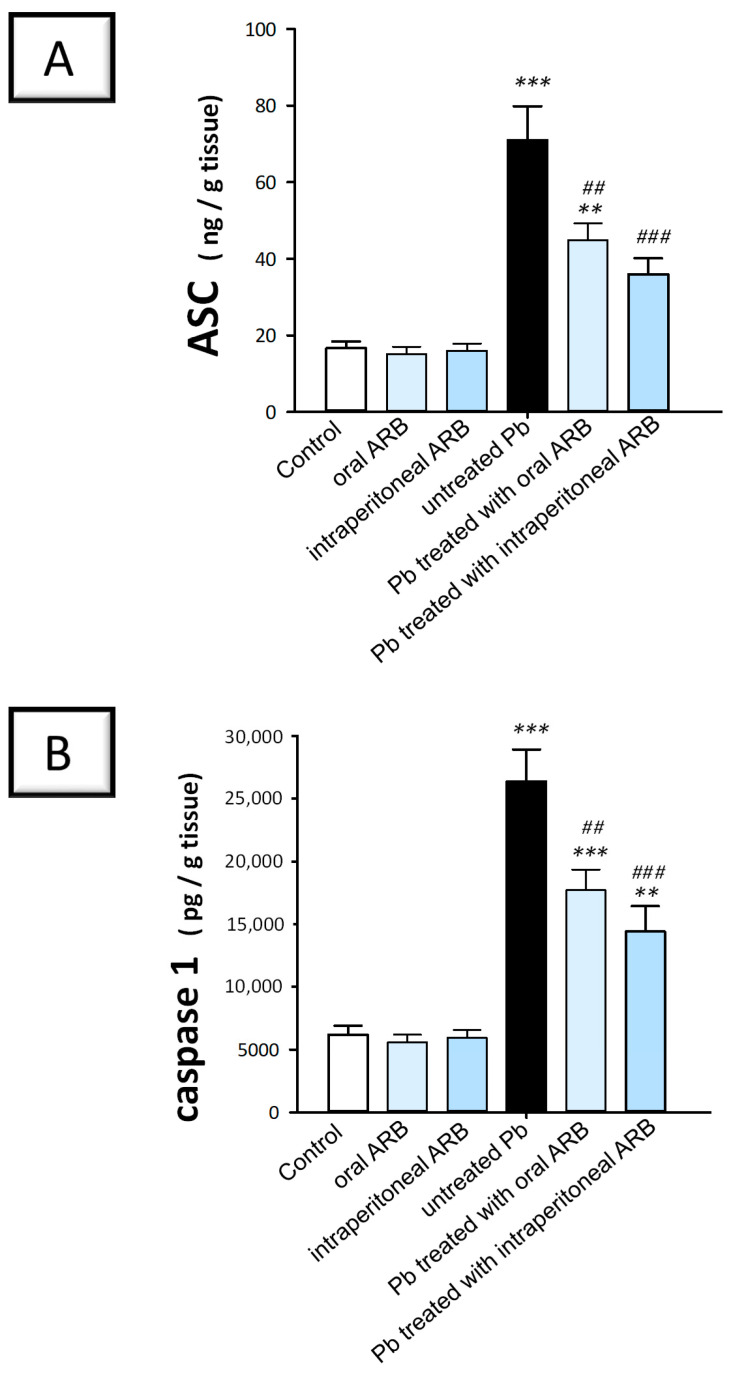
Modulatory effect of arbutin (ARB), administered either orally or via intraperitoneal injection, on testicular content of ASC and caspase 1 in lead (Pb)-evoked testicular damage in rats. (**A**) ARB lowered ASC in the testicular tissues of rats challenged with Pb. (**B**) Testicular tissues of rats challenged with Pb exhibited a reduction in caspase 1 content following the administration of ARB. Data represented as mean ± SEM (n = 6). Compared to the control group, significance was described by ** *p* < 0.0, or *** *p* < 0.001. Compared to the untreated Pb group, significance was described by ^##^ *p* < 0.01 or ^###^ *p* < 0.001 (as determined by Bonferroni’s test and one-way ANOVA).

To examine the potential effects of ARB on the Pb acetate-induced activation of the pro-inflammatory NLRP3/caspase-1/NF-κB pathway, we measured the levels of NLRP3, ASC, caspase-1, and NF-κBp65 in testicular tissues. NLRP3 is a key player in inflammatory response regulation. The stimulation of NLRP3 leads to caspase 1 activation with excessive pro-inflammatory cytokine production and NF-κB activation, culminating in testicular dysfunction and defective spermatogenesis [5,7]. The stimulation of NF-κB enhances the transcription of pro-inflammatory chemokines, cytokines, and adhesion molecules, further exacerbating the testicular inflammatory response [22,35]. As illustrated in Figure 6C and Figure 7A,B, rats challenged with Pb acetate exhibited a significant increase in testicular NLRP3, ASC, and caspase-1 levels of 244% (*p* < 0.001), 326.5% (*p* < 0.001), and 332% (*p* < 0.001), respectively, compared to control animals. The co-administration of ARB orally to Pb acetate-intoxicated animals triggered a significant decline in NLRP3, ASC, and caspase 1 by 52.7% (*p* < 0.001), 36.8% (*p* < 0.01), and 33.6% (*p* < 0.01), respectively. Likewise, the intraperitoneal administration of ARB significantly reduced (*p* < 0.001) NLRP3, ASC, and caspase-1 levels by 47.4%, 49.3%, and 46%, respectively, compared to the Pb acetate group.

**Figure 8:**
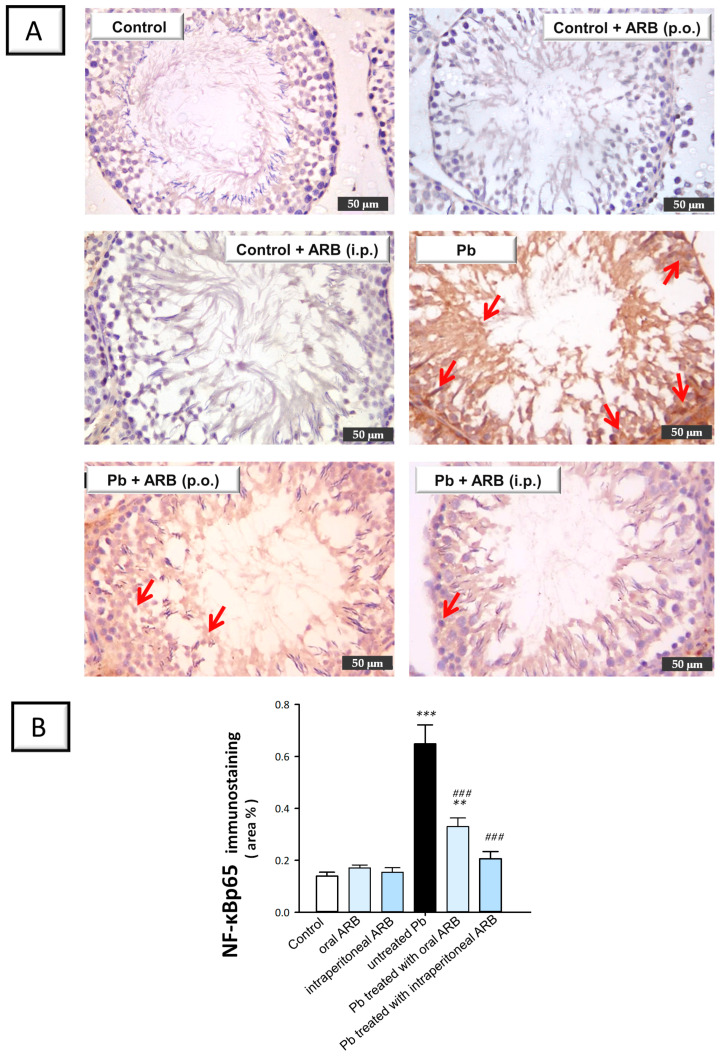
Ameliorative effect of arbutin (ARB), administered either orally or via intraperitoneal injection, on testicular protein expression of activated NF-κBp65 in lead (Pb)-evoked testicular damage in rats. The photomicrographs are representative of n = 6 tissues. (**A**) Photomicrographs of testicular tissues of all experimental groups. The immunoreactivity of NF-κBp65 was visualized in the tissues as a brown color developed by DAB chromogen (50-µm scale bar; positive staining is indicated by red arrows). (**B**) Quantification of NF-κBp65 expression in the testicular tissues. Data represented as mean ± SEM (n = 6). Compared to the control group, significance was described by ** *p* < 0.01 or *** *p* < 0.001. Compared to the untreated Pb group, significance was described by ^###^ *p* < 0.001 (as determined by Bonferroni’s test and one-way ANOVA).

We evaluated the testicular expression of activated NF-κBp65 using immunohistochemistry (Figure 8A). The testicular tissues of the control group and ARB-treated control groups (administered via oral and intraperitoneal routes) revealed negative NF-κBp65 immunoreactivity in the germinal epithelium and interstitial cells. In contrast, the Pb acetate group demonstrated extensive NF-κBp65 expression in the primary spermatogonia and Sertoli cells, with surrounding spermatids showing diffuse immunostaining. The staining was characterized by diffuse DAB signals in the background due to the marked pro-inflammatory response occurring in the testicular tissues. In the group co-treated with Pb and oral ARB, a marked decline in NF-κBp65 immunoreactivity was observed, confined to spermatids with minimal expression in other spermatogenic cells. Likewise, the group co-treated with Pb and intraperitoneal ARB showed weak NF-κBp65 immunoreactivity in the spermatids with minimal expression in spermatogenic cells. As depicted in Figure 8B, quantification of the immunohistochemistry data revealed that Pb acetate triggered a significant increase (*p* < 0.001) in testicular protein expression of activated NF-κBp65 of 367.2%. Conversely, the oral administration of ARB triggered a significant decline in NF-κBp65 testicular expression by 49.1% (*p* < 0.001). Likewise, the intraperitoneal administration of ARB exhibited a significant decrease in NF-κBp65 testicular expression of 68.2% (*p* < 0.001). Overall, the intraperitoneal injection of ARB to Pb acetate-intoxicated rats demonstrated a more pronounced suppression of ASC, caspase-1, and NF-κBp65 than the oral administration of ARB. According to these data, ARB’s suppression of the NLRP3/caspase-1/NF-κB axis was, at least partially, responsible for the amelioration of Pb acetate-evoked testicular damage.

### 2.7. Arbutin Stimulated the PK2/PKR2 Pathway and Curtailed Caspase 3 Expression in the Testes of Lead Acetate-Intoxicated Rats

**Figure 9:**
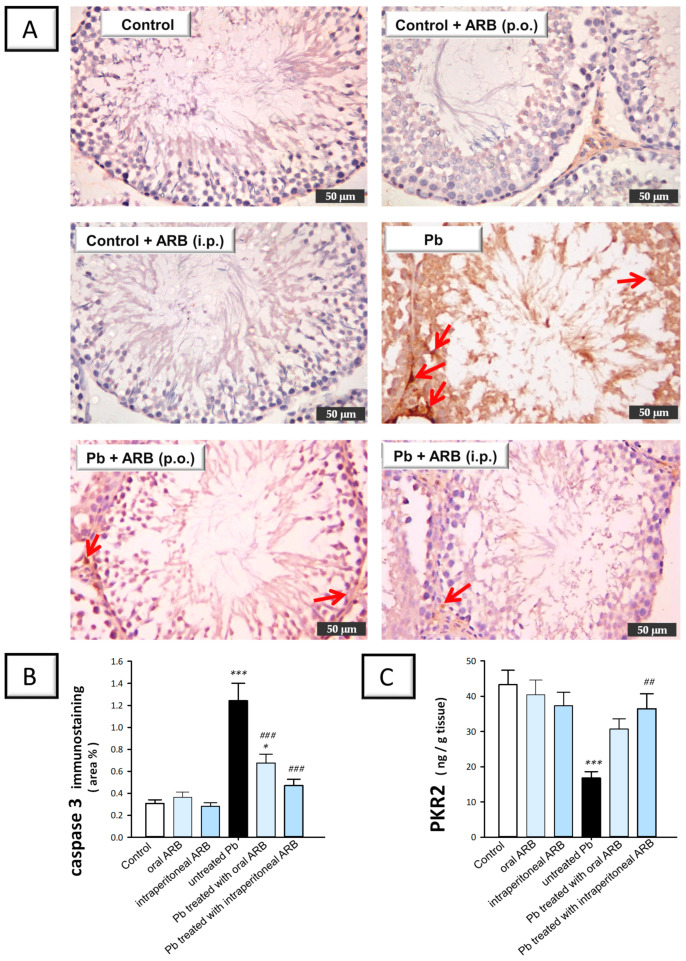
Ameliorative effect of arbutin (ARB), administered either orally or intraperitoneally, on testicular protein expression of caspase 3 and PKR2 in lead (Pb)-evoked testicular damage in rats. The photomicrographs are representative of n = 6 tissues. (**A**) Photomicrographs of testicular tissues of all experimental groups. The immunoreactivity of caspase 3 was observed in the tissues as a brown color developed by DAB chromogen (50-µm scale bar; positive staining is indicated by red arrows). (**B**) Quantification of caspase 3 expression in the testicular tissues. (**C**) ARB boosted the expression of PKR2 in the testicular tissues of rats challenged with Pb. Data represented as mean ± SEM (n = 6). Compared to the control group, significance was described by * *p* < 0.05 or *** *p* < 0.001. Compared to the untreated Pb group, significance was described by ^##^ *p* < 0.01 or ^###^ *p* < 0.001 (as determined by Bonferroni’s test and one-way ANOVA).

To investigate the potential effects of ARB on Pb acetate-induced PK2/PKR2 pathway dysfunction, we measured the levels of PK2, PKR2, and the pro-apoptotic signal caspase 3 in testicular tissues. The PK2/PKR2 is a cytoprotective pathway involved in the regulation of spermatogenesis and Leydig cell function. By acting through its receptor PKR2, PK2 elicits favorable testicular outcomes including proliferation, differentiation, and survival [13,14]. Conversely, caspase 3 is a pro-apoptotic executioner whose upregulation has been linked to impaired spermatogenesis and testicular dysfunction [13]. As illustrated in Figure 6D and Figure 9C, rats challenged with Pb acetate showed a significant decline in testicular PK2 and PKR2 by 77.6% (*p* < 0.01) and 61.2% (*p* < 0.001), respectively, compared to control animals. The co-administration of ARB orally to Pb acetate-intoxicated animals triggered a significant elevation in testicular PK2 by 230.8% (*p* < 0.05). Similarly, the intraperitoneal administration of ARB resulted in similar changes in these parameters by significantly elevating (*p <* 0.01) testicular PK2 and PKR2 by 307% and 117.2%, respectively.

As depicted in Figure 9A, the testicular expression of caspase 3 was evaluated using immunohistochemistry. The testicular tissues of the control group and ARB-treated control groups (administered via oral and intraperitoneal routes) showed negative caspase 3 immunoreactivity in the germinal epithelium of the seminiferous tubules, with weak reactivity in the interstitial cells in the control group that received ARB orally. Conversely, the Pb-acetate group demonstrated extensive caspase 3 expression in all germinal cells and interstitial cells, including Leydig cells. The staining was characterized by diffuse DAB signals in the background due to the marked apoptotic events occurring in the testicular tissues. In the group co-treated with Pb and oral ARB, a marked decline in caspase 3 immunoreactivity was observed, confined to interstitial cells, with weak expression in primary spermatogenic cells. Similarly, the group co-treated with Pb and intraperitoneal ARB showed weak caspase 3 immunoreactivity in interstitial cells, with minimal expression in the germinal epithelium. As depicted in Figure 9B, quantification of the immunohistochemistry data revealed that Pb acetate triggered a significant increase (*p* < 0.001) in testicular protein expression of caspase 3 of 304.3%. The oral administration of ARB triggered a significant decline in caspase 3 testicular expression by 45.5% (*p* < 0.001). Likewise, the intraperitoneal administration of ARB significantly lowered (*p* < 0.001) testicular caspase 3 expression by 62.2%. Overall, the intraperitoneal injection of ARB to Pb acetate-intoxicated rats demonstrated more pronounced effects on the PK2/PKR2 pathway and caspase 3 expression compared to the oral administration of ARB. According to these data, ARB’s augmentation of the PK2/PKR2 pathway and inhibition of caspase 3 expression was, at least partially, responsible for the amelioration of Pb acetate-evoked testicular damage.

## 3. Discussion

**Figure 10:**
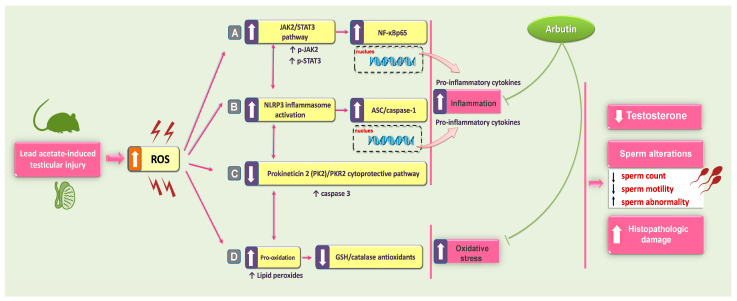
A summary of the current study’s outcomes and the molecular events through which arbutin mitigated Pb-triggered testicular dysfunction. The observed molecular changes of arbutin included the following. (A) Dampening the testicular inflammatory events by inhibiting the JAK2/STAT3 pro-inflammatory pathway in Pb-intoxicated rats. (B) Suppression of the inflammation-associated NLRP3/ASC/caspase-1 pathway in the testicular tissues. (C) Stimulation of the testicular cytoprotective PK2/PKR2 pathway, promoting the protection and survival of testicular germ cells. (D) Interference with the testicular prooxidant response and augmentation of antioxidant signals. In the figure, a solid arrow depicts activation, while a blunt arrow indicates inhibition.

The primary objective of the present study was to assess the potential of arbutin (ARB) to ameliorate the deleterious consequences of Pb toxicity on the testicular tissues of rats in vivo. The current results demonstrate that both oral and intraperitoneal administration of ARB significantly improved serum testosterone levels and sperm count/motility, indicating the mitigation of testicular dysfunction. Notably, the intraperitoneal administration of ARB at a lower dose improved the testicular pathological aberrations more effectively than orally administered ARB. At the molecular level, ARB inhibited testicular pro-oxidant and inflammatory events in rats intoxicated with Pb, culminating in the amelioration of testicular dysfunction and disrupted spermatogenesis. At least partly, the curbing of the NLRP3/ASC/caspase-1 axis and JAK2/STAT3 pathways and the activation of PK2/PKR2 were involved in the observed beneficial effects of ARB (Figure 10). Together, these findings suggest that ARB may be used as an adjunct agent in the management of Pb-induced testicular impairment. 

The present data revealed that ARB mitigated Pb-induced testicular injury and impaired spermatogenesis. In this regard, testicular tissue integrity, spermatogenesis, and sperm motility were significantly enhanced by ARB. It is suggested that the increased serum levels of testosterone as well as the improved quality of the semen likely reflect the competence of ARB to effectively counteract testicular damage and rectify the pituitary–gonadal axis. The disturbance of the pituitary–gonadal axis by Pb has been described in several reports. These findings also coincide with the previous literature that characterized disturbances of the pituitary–gonadal axis in several testicular pathologies [31,36,37]. Indeed, the observed increase in serum LH and FSH has been regarded as a compensatory response to testicular damage and diminished testosterone levels. This compensatory response attempts to stimulate the testicular tissues to generate testosterone and augment spermatogenesis [36]. At the molecular level, evidence revealed that excessive testicular pro-inflammatory and pro-oxidant events are associated with the disrupted secretion of LH and FSH [37]. In the context of spermatogenesis, we observed diminished serum testosterone in rats suffering from Pb intoxication, an event that undermined semen quality, including sperm count and motility, as previously reported by other investigators [3,4,6]. It is believed that these findings are likely triggered by Pb competence in crossing the blood–testis barrier, thereby disturbing the spermatogenesis process [3,4]. Evidence exists showing that Pb intoxication results in oligospermia, asthenospermia, and teratospermia due to the immature production of spermatogenic cells, delayed spermiation, and impaired epididymal functions [38]. 

The pathogenesis of Pb-induced testicular damage is regarded as multifactorial. The synergy among oxidative stress, inflammation, and apoptosis has been reported to mediate several testicular pathologies, including Pb-triggered testicular dysfunction [4,6,7,33]. Since oxidative stress mediates the pathogenesis of Pb-induced testicular injury, the current study aimed to examine whether ARB’s antioxidant features would attenuate the testicular damage. Interestingly, the current data revealed ARB’s competence to lower testicular lipid peroxidation in addition to augmenting reduced glutathione and catalase antioxidants. Previous studies have revealed that Pb instigates marked oxidative stress in the testes of rodents challenged with Pb [4,6,7], primarily by enhancing excessive ROS production, such as superoxide/hydroperoxide radicals and hydrogen peroxide [3]. Oxidative stress is believed to be one of the most significant causes of male infertility since it adversely affects both the structural and functional integrity of sperm [33]. There are several consequences of excess ROS production in the testis, including lipid peroxidation, inflammation, mitochondrial dysfunction, and sperm DNA fragmentation, culminating in testicular impairment and spermatogenesis failure [33]. In addition, the depletion of testicular Pb antioxidant moieties has been reported as a hallmark of its toxicity by binding to sulfhydryl groups or interfering with metal cofactors [6,7]. As a result, testicular oxidative stress impairs membrane integrity and protein function and results in excessive lipid peroxidation [4,6]. The observed ARB inhibition of testicular pro-oxidant events and augmentation of antioxidants have been envisioned as a privilege in the protection of testicular tissues. Generally, treatment modalities that dampen testicular redox disturbances and enhance antioxidant protection have been proven effective in combating multiple testicular pathologies [3,7,39,40]. The previous literature revealed that agents with antioxidant features can restore the balance in free radical production, thereby improving spermatogenesis and seminal plasma clearance [33,41]. A study of infertile men revealed that increased GSH and ascorbic acid antioxidants serve to halt ROS deleterious effects on sperm parameters [34]. Ample evidence revealed that ARB possesses marked antioxidant features. ARB lowers ROS levels directly by scavenging free radicals, such as hydroxyl radicals, or indirectly by augmenting the Nrf2-ARE pathway, thereby boosting cellular antioxidant potential [42]. In experimental studies, ARB has previously demonstrated evident antioxidant capacity by suppressing oxidative stress in a cyclophosphamide-induced hepatic injury model by dampening the levels of lipid peroxides and boosting catalase activity, culminating in favorable hepatic functional outcomes [19]. In a mouse model of ulcerative colitis, ARB suppressed myeloperoxidase activity, leading to improved intestinal pathology [18]. Moreover, boosting antioxidant defenses and curtailing ROS production interceded the beneficial effects of ARB in experimental lipopolysaccharide-triggered acute kidney injury [22] and cisplatin-evoked ovarian toxicity [21].

In this study, ARB’s potential for dampening testicular inflammation in rats with Pb intoxication was investigated. Interestingly, ARB demonstrated marked anti-inflammatory actions by suppressing several testicular pro-inflammatory signals, including the NLRP3/ASC pathway, and downregulating NF-κBp65 expression. Noteworthily, the current findings report for the first time ARB’s efficacy in modulating the NLRP3/caspase-1/NF-κB pathway. Notably, combating testicular inflammation is deemed a promising strategy for ameliorating several testicular pathologies [37,43]. In the context of testicular inflammation, the current study’s findings revealed an excessive pro-inflammatory response with the activation of the NLRP3/caspase-1/NF-κB pathway. Consistently, ample evidence demonstrated that inflammation plays an important role in Pb toxicity [44]. In the presence of excessive ROS, the redox-sensitive transcription factor NF-κB initiates the transcription of several pro-inflammatory cytokines [4,6,7]. The crosslink between oxidative stress and exaggerated inflammatory response following Pb exposure has been described earlier [4,6,8]. At the cellular level, ROS instigates the phosphorylation/degradation of the inhibitory subunit IκB, resulting in NF-κBp65 liberation. Following its nuclear translocation, NF-κBp65 interacts with the promoter region of pro-inflammatory cytokine genes, triggering excessive pro-inflammatory cytokine production, like TNF-α and IL-1β [22,35]. The current study also revealed the upregulated expression of testicular NF-κBp65 alongside NLRP3, ASC, and caspase 1 in response to Pb intoxication. In the context of inflammasomes, there are three defined components that compose the NLRP3 inflammasome. One is the NLRP3, which is the ROS sensor; another is the ASC subunit, which is the adapter component; and the third component is the caspase effector [5,45]. A major site of NLRP3 expression is the Sertoli cells in rodents and primates, along with the peritubular cells in humans, responsible for testicular immune surveillance. Hence, the function of these cells may be closely associated with NLRP3 [5,33]. Regulating NLRP3 inflammasomes involves a two-step process [5,33]. First, ROS serves as a “priming” stimulus, which stimulates the upregulated expression of the three components before they are assembled. Following the priming signal, there is an activation signal, which results from ROS production, mitochondrial DNA release, and lysosomal damage/dysfunction. The activation signal triggers NLRP3 complex assembly, leading to caspase 1 stimulation [5,7]. Evidence revealed that inflammasome activation and excessive ROS production negatively affect the spermatogenesis process, leading to sperm DNA fragmentation and motility dysfunction [33]. In a rodent model of abnormal semen quality following a spinal cord injury, the interaction between NLRP3 and lipid peroxidation was described. Inflammasome activation and increased lipid peroxidation directly correlate with decreased semen quality [33,46]. The interplay between oxidative stress and NLRP3 has been characterized, whereby oxidative stress triggers NLRP3 inflammasome activation and excessive cytokine production, which induces testicular leucocyte influx. The latter event spikes ROS production and overwhelms testicular antioxidant capacity, culminating in spermatogenic failure and testicular impairment [5,7,33]. Considering the observed ARB’s attenuation of testicular inflammatory events and inhibition of the NLRP3/Caspase-1/NF-κB axis, several reports have characterized similar findings. In a rat model of hepatotoxicity, ARB was proven to dampen the expression of hepatic NF-κBp65 and pro-inflammatory cytokines, leading to curtailed hepatic injury [19]. The anti-inflammatory impact of ARB was also shown in lipopolysaccharide-stimulated BV2 microglial cells. In the study, ARB curbed NF-κB nuclear translocation, inhibited leukocyte adhesion, and downregulated several pro-inflammatory genes, pointing to its efficacy in dampening inflammatory conditions [35]. The work by Zhang et al. [22] provided evidence of ARB inhibiting inflammation and improving renal functional outcomes by lowering pro-inflammatory cytokines and limiting the protein expression of activated NF-κBp65 in lipopolysaccharide-induced acute kidney injury. In the same regard, evident anti-inflammatory actions of ARB have interceded in its ameliorative effects in rodents with ulcerative colitis [18] and cisplatin-triggered ovarian toxicity [21].

We further examined the potential of ARB to dampen the JAK2/STAT3 pro-inflammatory pathway in the testicular tissues of rats intoxicated with Pb. In addition to suppressing the NLRP3/ASC pathway, the present findings revealed that ARB also effectively inhibited the JAK2/STAT3 pathway, a pro-inflammatory signal known to play a pivotal role in the progression of several testicular pathologies [9,12]. As a redox-sensitive kinase, JAK2 is stimulated by ROS overshooting, leading to the initiation and progression of male reproductive dysfunction [9]. In ischemia–reperfusion testicular injury, activation of the JAK2/STAT3 pathway has been associated with spermatogenic arrest and the weakened expression of Sertoli cell junctions [9,10]. In the same experimental paradigm, the JAK2/STAT3 signaling pathway has also been implicated in ROS-induced DNA damage in germ cells [9]. Furthermore, JAK2/STAT3 activation has been reported to mediate testicular impairment and sperm aberrations in rheumatoid arthritis-associated testicular damage [12]. At the molecular level, the JAK2/STAT3 signaling pathway is downstream of several ligand-binding signals, such as pro-inflammatory cytokines. Upon activation through phosphorylation, STAT3 forms a dimer that migrates to the nucleus to activate target genes encoding chemokines and cytokines, perpetuating the inflammatory cycle and associated testicular damage [11,12]. In macrophages, phosphorylated STAT3 enhances NF-κB transcription, resulting in sperm abnormalities and diminished testosterone production [47]. Remarkably, the findings of the present study revealed JAK2/STAT3 activation with the enhanced expression of activated NF-kBp65 in the testicular tissues of Pb-challenged animals. In vivo studies and bioinformatic analyses demonstrated a correlation between hypoxia and oligozoospermia and the enhanced testicular expression of JAK proteins [12,48]. In testicular dysfunction, including testicular ischemia/reperfusion injury, the JAK/STAT pathway is known to be activated by oxidative stress-triggered apoptosis [10,12]. ARB administration evidently inhibited testicular JAK2/STAT3 by dampening the phosphorylation of its protein signals. Indeed, the inhibition of JAK2 phosphorylation has been linked to preserved spermatogenesis and enhanced seminiferous tubule integrity [12], likely via dampening ROS generation/lipid peroxidation, suppressing apoptosis, and enhancing antioxidant signaling. 

The current work also aimed to explore whether the beneficial outcomes of ARB are linked to potential PK2/PKR2 pathway modulation in the testicular tissue of rats intoxicated with Pb. The present findings revealed that ARB effectively enhanced the testicular cytoprotective PK2/PKR2 pathway. In testicular tissue, PK2 proteins are highly expressed by primary spermatocytes, as evidenced by accumulating evidence. The PK2/PKR2 pathway plays a crucial role in fertility and sexual development in male rodents since mice with PK2 and PKR2 knockout failed to secrete gonadotropin-releasing hormones [13,14]. Previous reports have demonstrated the downregulated expression of PK2 and PKR2 in diverse testicular pathologies, including diabetes mellitus-associated testicular damage [13]. Noteworthily, the observed Pb-induced PK2 downregulation contradicts the findings reported by Li et al. [49], which revealed elevated PK2 expression in an experimental model of autoimmune orchitis. Disparities may be a result of the differences in the experimental model, the severity of the testicular injury, and the duration of exposure to the insulting agent. Interestingly, the ARB-induced amelioration of testicular damage was associated with an upregulated PK2/PKR2 pathway. Indeed, PK2/PKR2 is essential for testicular germ cell protection and survival through inhibiting oxidative stress and apoptosis [13]. Moreover, PKR2 has been characterized as blocking the assembly of the NLRP3 inflammasome, preventing its activation [13,50]. According to the literature, a modality that can activate the PK2/PKR pathway in testicular pathologies has the potential to exert positive effects on testicular function as well as spermatogenesis [13,14]. In experimental models, PK2 exhibits anti-inflammatory and antiapoptotic actions, with beneficial outcomes against experimental cardiomyopathy [51], hippocampal damage [50], and neuronal degeneration [52]. In the same regard, the observed blockade of caspase 3 by ARB is consistent with a previous study demonstrating that ARB can counteract apoptotic events by limiting pro-apoptotic signals, including caspase 3, and enhancing pro-survival pathways in LPS-evoked kidney injury and LPS-stimulated NRK-52e cells [22]. 

## 4. Materials and Methods

### 4.1. Drugs and Chemicals

Arbutin (ARB; CAT # A4256) and lead acetate (as trihydrate; CAT # 32307) were purchased from Sigma-Aldrich (St. Louis, MO, USA). The catalog number and supplier’s names for kits were described under each assay. A high grade of purity was acquired for all remaining chemicals.

### 4.2. Experimental Animals

We purchased male albino rats of the Sprague–Dawley strain (150–200 g body weight) from the animal house of the Egyptian Drug Authority (EDA). Before experimentation, the animals were housed in plastic cages and allowed to acclimate for one week in the animal house. Rats were kept under controlled experimental conditions, including a 12-hour light/dark cycle, 20 ± 3 °C temperature, and 40% relative humidity. The animals had free access to water and a standard rodent pellet diet (20% protein, 5% fat, 75% carbohydrate, 5% fiber, and 10% moisture). To minimize stress and other factors affecting metabolic parameters, the animals were carefully handled by experienced investigators. To this end, we followed the guidelines published by the National Institutes of Health on Laboratory Animal Care and Use (NIH Publication No. 85-23, revised 2011). The Animal Experiment Ethics Committee at the EDA endorsed the current study protocol (ethical reference no. NODCAR/I/12/2022).

### 4.3. Study Design

**Figure 11:**
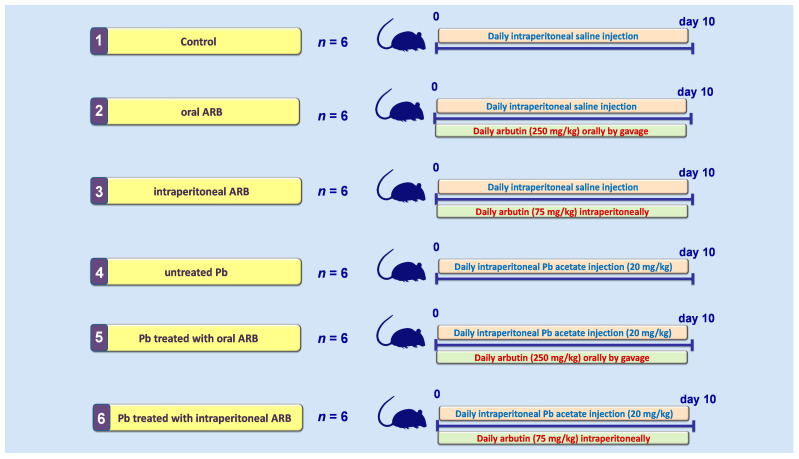
Summary of the experimental design. ARB, arbutin; Pb, lead.

Based on the experimental design presented in Figure 11, 36 rats were randomly distributed into six groups (n = 6 rats per group) after a week of acclimatization. The following procedures were applied: **Group I (control gp.)**: received daily intraperitoneal injection of saline for 10 consecutive days. **Group II (control treated with oral ARB)**: received daily intraperitoneal saline injections in addition to oral gavage of arbutin (250 mg/kg/day; 2 h after saline injection) for 10 days. **Group III (control treated with intraperitoneal ARB)**: received daily intraperitoneal saline injections in addition to intraperitoneal injections of arbutin (75 mg/kg/day; 2 h after saline injection) for 10 days. **Group IV (untreated Pb):** received intraperitoneal injections of Pb acetate (20 mg/kg/day) for 10 consecutive days. **Group V (Pb treated with oral ARB):** received intraperitoneal Pb acetate injections (20 mg/kg/day) and oral gavage of arbutin (250 mg/kg/day; 2 h after Pb acetate injection) for 10 days. **Group VI (Pb treated with intraperitoneal ARB):** received intraperitoneal Pb acetate injections (20 mg/kg/day) and intraperitoneal injections of arbutin (75 mg/kg/day; 2 h after Pb acetate injection) for 10 days. 

Previous research reports guided us in selecting the current experimental protocol for Pb-induced testicular dysfunction [6,24,25]. In the current experimental design, ARB was administered orally [53] and intraperitoneally at a lower dose [27,53,54] to compare the efficacy of both routes in mitigating Pb acetate-induced testicular dysfunction. This approach is in line with the previous literature [27,53,54]. Generally, the oral route is regarded as more convenient for ARB administration in the experimental setting. Intraperitoneal administration is associated with higher bioavailability due to limited first-pass hepatic metabolism, culminating in minimal adverse effects [23,26]. More specifically, the used dose of ARB administered via the intraperitoneal route is consistent with previous reports that confirmed the competence of ARB to mitigate experimental models of monosodium L-glutamate-induced cognitive decline and neurotoxicity [27], 1-methyl-4-phenyl-1,2,3,6-tetrahydropyridine (MPTP)-induced Parkinson’s disease [54], and carbon tetrachloride-induced hepatic damage [53]. The oral dose of ARB was chosen based on earlier research that showed its efficacy in ameliorating experimental carbon tetrachloride-induced hepatic damage [53].

In the current study’s design, we used a protective regimen for ARB administration, where ARB was co-administered with Pb injection. This approach coincided with several previous reports that investigated the efficacy of different agents for ameliorating Pb-induced testicular dysfunction [6,24,25]. Given Pb’s widespread existence in human industries, including metal smelting, painting, glass-blowing, battery repair, and welding [1,4,6,7], the protective approach was preferred to simulate human exposure conditions. This emphasizes the principle that prevention is superior to treatment.

Following thiopental anesthesia (30 mg/kg; i.p.) [55], rats were euthanized by cervical decapitation at the end of the study. A retro-orbital blood sample was collected for serum isolation. The testes were rapidly collected and appropriately processed for subsequent biochemical, molecular, histological, and immunohistochemical analyses.

### 4.4. Determination of Sperm Parameters

The method described by Bearden and Fuquay [28] was used for seminal examination. In a sterile watch glass, the content of the epididymis of each sample was collected in a sterile watch glass after cutting the cauda epididymis and gentle squeezing. Then, the epididymis was collected in a sterile watch glass, and sperm motility, count, and abnormalities were determined [29]. On a glass slide, a small droplet of semen was mixed with a solution of 2.9% sodium citrate and kept at a temperature of 37 °C. Under a light microscope (10×); several fields were examined and the percentage of sperm motility was recorded. Sperm counting was achieved by applying a drop of diluted semen between the cover and the hemocytometer slide under a light microscope with a 40-× lens. The Eosin–Nigrosin stain was combined with a droplet from the seminal fluid to evaluate sperm anomalies. To this end, the films were analyzed at random per slide under a 40× lens, and abnormal sperm % was calculated.

### 4.5. Determination of Sex Hormones

To measure the content of the luteinizing hormone (LH) and follicle-stimulating hormone (FSH) in serum, specific ELISA kits were purchased from Elabscience Biotechnology, as directed by the vendor’s protocol (CAT # E-EL-R0026 (LH) and CAT # E-EL-R0391 (FSH); Houston, TX, USA). For the measurement of serum testosterone and testicular acid phosphatase (ACP) activity, specific kits were procured from Cusabio Technology and BioVision, respectively, as directed by the vendor’s protocol (CAT # CSB-E05100r; Houston, TX, USA for testosterone and CAT # K411-500; Milpitas, CA, USA for ACP for ACP). 

### 4.6. Determination of Testicular Redox Milieu

To measure the content of lipid peroxides in the testicular tissues, the colorimetric assay established by Ohkawa et al. [56] was applied. The content of reduced glutathione (GSH) in the testicular tissue was determined by spectrophotometry, as described by Beutler et al. [57]. To determine testicular catalase activity, the assay established by Aebi [58] was applied. 

### 4.7. Assessment of p-STAT3, ASC, Caspase 1, and PKR2

To measure the content of phosphorylated STAT3 (CAT # PEL-Stat3-Y705; RayBiotech, Norcross, GA, USA) and ASC (CAT # EKU11381; Biomatik; Wilmington, DE, USA) in the testicular tissues, specific ELISA kits were used, as directed by the vendor’s protocol. Moreover, the measurement of the content of caspase-1 and PKR2 in testicular tissue was conducted using specific kits (CAT # E-EL-R0371; Elabscience Biotechnology, Houston, TX, USA for caspase 1 and CAT # abx539130; Abbexa LLC, Houston, TX, USA for PKR2), as directed by the vendor’s protocol. 

### 4.8. Western Blot Analysis

Further assessment of the testicular injury was applied by detecting testicular NLRP3, PK2, and p-JAK2 by Western blot analysis. The testicular tissue was homogenized using a polytron homogenizer, following the mixing of 50 mg of testicular tissue with cold lysis buffer (NP40 (1%), SDS (1%), Tris-HCl; pH 8.0, sodium deoxycholate (0.5%), NaCl, and phenylmethylsulfonylfluoride), as described earlier [59]. Proteins were separated by SDS-PAGE and the resolved proteins were transferred to nitrocellulose membranes (Bio-Rad Hercules, CA, USA). After blocking the membranes, specific antibodies were incubated at 4 °C overnight against NLRP3 (CAT # ab263899; Abcam, Waltham, MA, USA; 1:1000), PK2 (CAT # ab87360; Abcam, Waltham, MA, USA; 1:1000), or p-JAK2 (CAT # 3776; Cell Signaling Technology, Danvers, MA, USA; 1:1000). Following a thorough wash of membranes, the horseradish peroxidase-conjugated secondary antibody incubation was conducted. The detection and visualization of target proteins were performed using a commercial kit (enhanced chemiluminescence) purchased from Amersham, as specified by the vendor (Arlington Heights, IL, USA). Molecular Analyst Software (version 1.6.3) was used for densitometric quantification, as instructed by the manufacturer (Bio-Rad, Hercules, CA, USA). 

### 4.9. Histology

A formaldehyde solution (10%) was used to fix the testicular specimens and the samples were then processed to form paraffin cubes. With the help of a microtome, tissue blocks made from paraffin wax were cut into 4 µm thick slices. A histopathological examination of the tissue sections was applied using light microscopy (Leica Microsystems GmbH, Wetzlar, Germany) after staining with hematoxylin and eosin (H-E) [29]. A blinded image analysis was conducted to limit bias.

For the quantitative examination of spermatogenesis, Johnsen scoring was applied [32]. In this context, spermatogenesis was evaluated on a scale of 1 (complete impairment of spermatogenesis) to 10 (normal/complete spermatogenesis) in the seminiferous tubules. Complete spermatogenesis was scored as 10, disorganized spermatogenesis with the presence of many spermatozoa was scored as 9, disorganized spermatogenesis with the presence of few spermatozoa was scored as 8, the absence of spermatozoa and existence of many spermatids was scored as 7, the presence of only a few spermatids was scored as 6, the absence of spermatozoa/spermatids and existence of many spermatocytes was scored as 5, the presence of only a few spermatocytes was scored as 4, the presence of only spermatogonia was scored as 3, a lack of germ cells was scored as 2, and the absence of germ cells and Sertoli cells was scored as 1.

### 4.10. Immunohistochemistry (IHC)

We applied IHC as an effective tool for examining the expression of NF-κBp65 and caspase 3 proteins and their localization within testicular tissues. As part of these procedures, tissue sections were dewaxed and rehydrated, and antigens were removed from them by boiling them in citrate buffer (pH 6.0). Hydrogen peroxide (3%) was applied for 20 min to quench tissue endogenous peroxidase activity. Then, 5% bovine serum albumin was used as a blocking agent in a humidified chamber to avoid the non-specific binding of antibodies to tissue proteins. Next, 4 µm sections of testicular tissues were immunostained with anti-caspase-3 (CAT # 9662; Cell Signaling Technology, Danvers, MA, USA; 1:400) or anti-NF-κBp65 (CAT # 51-0500; ThermoFisher Scientific, Cambridge, MA, USA; 1:100) primary antibodies for 90 min, as previously characterized [60]. After this, the samples were washed and treated for 30 min with secondary antibodies that were HRP-labeled. A diaminobenzidine (DAB) kit was used for staining the target proteins (ScyTek Laboratories, Inc., Logan, UT, USA) and counterstaining was applied using hematoxylin. Images of tissue sections were captured using a digital imaging system attached to a light microscope (Meiji Techno Co., Saitama, Japan; Model MX5200L). Photographs were taken of six non-overlapping fields per specimen. For data analysis, the ImageJ Fiji program (version 1.2; https://imagej.net/Fiji/Downloads; accessed on 9/01/2023) was used to perform deconvolution and downstream analysis (NIH, Bethesda, MD, USA). In all groups, area percentages for caspase-3 and NF-κBp65 immunoreaction were measured at a magnification of ×400. A blinded image analysis was conducted to limit bias. 

### 4.11. Statistical Analysis

Statistical calculations were applied using GraphPad Prism (version 7; San Diego, CA, USA). For the purpose of determining the normality of values, a Shapiro–Wilk test was applied. Values were displayed as the mean ± SEM (parametric results). We conducted group comparisons using a one-way analysis of variance (ANOVA). To compare different groups, the post-hoc Bonferroni’s test was then selected. Analysis of the significant differences among groups for Johnsen’s scores (non-parametric) was conducted using Kruskal–Wallis and Dunn’s tests. Medians and interquartile ranges were presented for these values. The minimum significance threshold was set at *p* < 0.05. 

## 5. Conclusions

In summary, the current findings demonstrate the role of ARB’s anti-inflammatory and antioxidant properties in improving the testicular impairment prompted by Pb. In this regard, ARB restored the imbalance between excessive ROS production and the pro-inflammatory NLRP3/caspase-1 and JAK2/STAT3 cascades and augmented the cytoprotective PK2/PKR2 pathway, thereby improving spermatogenesis and semen quality. Thus, we suggest using ARB as an adjunct agent to combat the testicular impairment caused by Pb. Further studies are needed to provide detailed insights into ARB’s therapeutic efficacy and the molecular mechanisms implicated in ARB’s protection, particularly ROS-induced damage, including peroxidation, nitration, and carbonylation processes. Moreover, examining animals’ metabolic profiles and sperm DNA integrity would add to our understanding of ARB’s protection. Clinical research is warranted to explore ARB’s potential competence in testicular injury management.

## Data Availability

Data are contained within the article.
